# Neurocognitive Signatures of Naturalistic Reading of Scientific Texts: A Fixation-Related fMRI Study

**DOI:** 10.1038/s41598-019-47176-7

**Published:** 2019-07-23

**Authors:** Chun-Ting Hsu, Roy Clariana, Benjamin Schloss, Ping Li

**Affiliations:** 1 Department of Psychology and Center for Brain, Behavior, and Cognition, Pennsylvania State University, University Park, PA 16802 USA; 2 Kokoro Research Center, Kyoto University, 46 Yoshidashimoadachi-cho, Sakyo-ku, Kyoto 606-8501 Japan; 3 Department of Learning and Performance Systems, Pennsylvania State University, University Park, PA 16802 USA

**Keywords:** Language, Reading, Human behaviour

## Abstract

How do students gain scientific knowledge while reading expository text? This study examines the underlying neurocognitive basis of textual knowledge structure and individual readers’ cognitive differences and reading habits, including the influence of text and reader characteristics, on outcomes of scientific text comprehension. By combining fixation-related fMRI and multiband data acquisition, the study is among the first to consider self-paced naturalistic reading inside the MRI scanner. Our results revealed the underlying neurocognitive patterns associated with information integration of different time scales during text reading, and significant individual differences due to the interaction between text characteristics (e.g., optimality of the textual knowledge structure) and reader characteristics (e.g., electronic device use habits). Individual differences impacted the amount of neural resources deployed for multitasking and information integration for constructing the underlying scientific mental models based on the text being read. Our findings have significant implications for understanding science reading in a population that is increasingly dependent on electronic devices.

## Introduction

Reading expository texts remains a primary means for students to acquire scientific knowledge. Learning from such texts crucially depends on the reader’s ability to construct a mental representation that can maximally capture the knowledge structure (KS) inherent in the text. The text’s KS reflects the author’s conceptual knowledge associations, and the text KS interacts with the reader’s cognitive abilities that together impact the learning outcome of the reader’s representation of the scientific knowledge after reading^1,2^. The current study is designed to examine this interaction, specifically how the KS of the text (referred to as *textual KS* henceforth) interacts with the individual reader’s abilities in executive function and his or her reading habits (including electronic device usage). To understand this complex interaction properly, we studied expository science text reading at both the behavioural and the neurocognitive level, combining methods of network analyses of the reading material with statistical analyses of the data collected from self-paced naturalistic reading.

Until now, neurocognitive studies of reading comprehension have focused on narrative texts, and the major theories in the field have also been based on analyses of narrative texts^3^. When reading expository texts, the reader’s task is to identify the different possible relationships among often quite abstract concepts. These relationships can be correlational, temporal sequential, causal, or hierarchical, and can exist between pairs or clusters of concepts. Comprehension of these relationships in the text (in addition to understanding the meaning of words and facts about the world) is thus key to the reader’s success in expository text comprehension^4^.

An influential model of reading comprehension, the Construction-Integration model^5,6^, suggests that text comprehension is organized in cycles, roughly corresponding to short sentences or phrases^7,8^. The *construction* process takes place early in the cycle, in which the reader forms concepts and propositions from the linguistic input. Later in the cycle, the *integration* process establishes an elaborated propositional representation that is internally coherent and reasonably consistent with the discourse context and with the reader’s world knowledge. However, this early construction vs. late integration dual-stage processing view has been recently challenged by the view of parallel integration of information at different levels and scales. For example, Kuperberg and Jaeger^9^ proposed that during reading, predictive candidates are activated before the incoming new information is processed (i.e., top-down processing). The predictive pre-activation encompasses multiple levels of representations including syntactic, semantic, phonological, orthographic and perceptual. From the perspective of memory, Hasson *et al*.^10^ argued that all cortical circuits are involved in information accumulation in a hierarchical organization. The primary perceptual-motor systems have short process memory, while the higher order areas such as temporoparietal junction, angular gyrus, and medial prefrontal cortex have long process memory. The primary process areas are modulated by the fronto-parietal network of attentional control, while the higher-order areas by the medial temporal lobe (hippocampal) circuit of binding and consolidation. Therefore, the construction and integration processes might not be temporally dissociable, and instead, it is the brain regions that integrate information at different time scales of memory processes that should and can be empirically differentiated, such as distinct neural networks involved in the integration of local and global contexts^11^.

For expository texts, the information integration process encompasses analogous transfer^12,13^ or knowledge revision^3^, where updated situation models^14^ or mental models^15^ are generated. The extent to which the reader generates an appropriate situation model, an integrative mental representation of the text knowledge, depends on the one hand on how the knowledge is conveyed to them (e.g., text properties) and on the other, the reader’s cognitive abilities, including abilities to retain information in memory, sustain attention during reading, and formulate abstract concept relations (i.e., reader characteristics). These knowledge-specific and reader-specific characteristics can be examined, as in this study, under the umbrella of textual KS, and executive function and reasoning abilities, respectively.

## Knowledge Structure as Network Maps

Textual KS refers to how concepts/units of information are organized in an expository text^16^. Kintsch and van Dijk first proposed this idea using graphs to represent the network of coherent propositions^8^ in texts and Ferstl and Kintsch were among the first to apply network measures to estimate a reader’s situation model^17^. Network maps are one common explicit visual representation of KS, which consist of pairs of concepts (represented as nodes) joined by link lines (represented as edges) indicating relationships between pairs of concepts. This type of KS representation is now well established in the literature (see Kinchin *et al*.^18^ for discussion). In this study, we extracted the *textual KS* as network maps according to Clariana^19^. This process involves several steps as described in the Methods section.

**Figure 1:**
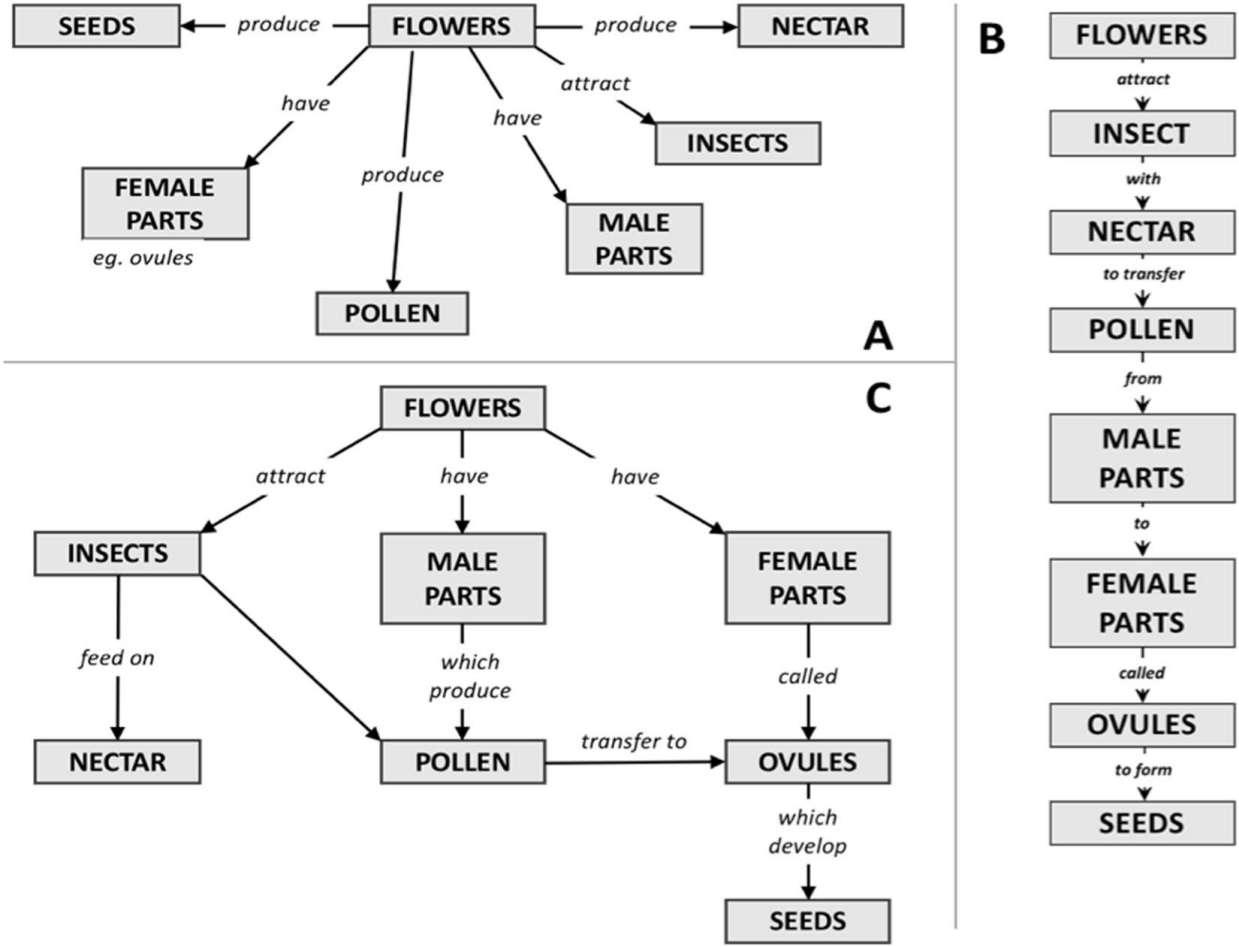
Three main concept map structures (reproduced from Kinchin *et al*.^18^). (**A**) Spoke **–** a radial structure in which all the related aspects of the topic are linked directly to the core concept, but are not directly linked to each other. (**B**) Chain – a linear sequence of understanding in which each concept is only linked to those immediately above and below. (**C**) Net – a highly integrated and hierarchical network

Among numerous network metrics that could be derived, centrality has been proposed as one of the most basic and pragmatic ways to describe network maps^20^. The centrality of a node in a network indicates the relative importance of that node in relation to all other nodes, and this measure has been used as a way to quantify the structure or shape of concept maps^21^. For example, Kinchin *et al*.^18^ categorized concept maps in terms of the network topologies of spoke, chain, and net (Fig. 1), and a major discriminating criterion was graph centrality. The *spoke* type concept map has a large graph centrality value, meaning that one central concept is connected to a large proportion of all other concepts. It represents a KS of simple associations, with no hierarchy and little integration of concepts. On the opposite end of this spectrum is the *chain* type map, which has a small graph centrality value, representing a sequential KS of isolated conceptual understanding with few associations among the concepts. Such concept maps are susceptible to “meltdown” from a single broken link, and are unlikely to then reorganize. In between these two extreme types is the *net* type map that has a medium graph centrality value, representing a KS of higher integrity with several levels of hierarchy and with complex interactions between levels. Reorganization of KS by incorporating would-be knowledge is well supported in maps of the net type, and missing links can more easily be compensated with redundant paths. For example, four behavioural studies^22–25^ that have considered this relationship have reported an ‘inverted U-shape function’ (as suggested by Rikers *et al*.^26^) for network graph centrality (abscissa, x-axis) and post-test measures (ordinate, y-axis), with the function’s maximum agreeing with the network graph centrality of the experts’ network. In sum, the shape of the network (spoke, net, chain) is related to the degree of conceptual integrity in the KS, and can be represented by different centrality scores.

Applying this logic in this study, we consider network maps with medial graph centrality values to represent near optimal *textual KS*, whereas maps with (extremely) high or low graph centrality values represent sub-optimal textual KS. Specifically, we use the maximal betweenness centrality (MBC)^27^ value, the highest betweenness centrality values of all nodes in a network to describe the characteristic of each textual network map. Also, we use the quadratic terms of the mean-centred/normalized MBC values as a measure of textual KS optimality: higher quadratic centrality values (further away from zero) indicate sub-optimal KS, while lower quadratic centrality values (closer to zero) indicate more optimal KS; the adoption of the quadratic terms is based on several established observations in previous studies^22–25^. Note that the definition of sub-optimal text here does not automatically mean a ‘bad’ or ‘incoherent’ text. Rather, the text KS typology depends on the nature of the domain knowledge; for example, optimal KS here refers to texts that have a KS structure hierarchically organized as central versus peripheral concepts.

## Executive Function, Reasoning, and Text Comprehension

Text comprehension results from how executive functions and analogical reasoning are employed by the reader to process the textual information^28^. Executive functions consist of a set of dissociable processes that coordinate cognition and facilitate goal-oriented behaviour^29^. Follmer’s^30^ meta-analysis showed positive correlations between reading comprehension and the following components of executive function: working memory, shifting, inhibition, and sustained attention and monitoring. In particular, working memory is needed to maintain and update textually relevant information on a constant basis, thereby facilitating the reader’s development of a mental representation of the text^31^. In the current study, we assess these four important components of executive functions through widely used standardized tests, the ‘attention network test’^32^ for measuring shifting and inhibition and the ‘letter-number sequencing test’^33^ for measuring working memory.

Another cognitive ability, analogical reasoning^12^, also significantly affects reading comprehension, although it is less well examined as compared with executive function. In analogical transfer^12^, the existing KS serves as the source or reference, and the newly formed textual KS is the target in the analogical process. For example, in chemistry classes, the solar system is often used as the source/referential analogy when explaining atomic structure (target concept). Analogical reasoning is also involved in reading when readers revise or update existing KS based on the new textual KS through reading comprehension. They compare and detect any inconsistency between the two, and if successful, further convert and incorporate the text information into prior knowledge for future use^3^. In this study, we assess analogical reasoning by using a standardized test, the Raven’s Progressive Matrices^34^.

Although no neuroimaging work has examined text comprehension based on the reader’s analogical reasoning ability, there is a sizable literature on the neural correlates of analogical reasoning. An aggregated meta-analysis of 7 studies^35^ showed neural correlates of semantic analogy in left IFG, MFG, frontopolar cortex (FPC), dorsolateral prefrontal cortex (DLPFC), and bilateral caudate heads. In particular, the left FPC is also involved in analogical reasoning of matrix problem tasks (e.g., based on Raven’s task) and visuospatial domains. This finding is consistent with the proposal that FPC is critical for integrating the outcomes of separate cognitive operations to facilitate long-term goal oriented behaviour^36,37^.

## Electronic Device and Reading

Individual differences also exist in areas other than executive function and analogical reasoning, and in a recent study, Follmer *et al*.^38^ investigated how different reading background variables relate to the individual’s reading comprehension of STEM (Science, Technology, Engineering, Mathematics) texts. Using a large sample of Mechanical Turk participants, they showed that STEM text comprehension was negatively correlated with reported frequency of reading on electronic devices (e.g., smartphones, tablets, computers) as well as with reported frequency of non-reading behaviour on electronic devices (e.g., watching television). At the same time, STEM text comprehension was positively correlated with self-reported level of reading attitudes and preferences (e.g., enjoyment of challenging books, learning difficult things via reading). These disturbing findings provided initial evidence of how the emerging electronic reading habits may fundamentally alter readers’ comprehension of expository scientific texts^39^.

Previous studies have investigated the effect of paper vs. screen-based reading comprehension (see Sidl *et al*.^40^ for a review), with findings indicating that reading on a screen, as compared with reading on paper, may lead to worse performance (or more reading time for achieving the same level of performance). Such discrepancies have been attributed to aspects of the technology such as visual fatigue and less convenient navigation, and also to the impact of electronic devices on metacognitive processes (e.g., overconfidence and reduced self-regulation and monitoring). However, recent studies have taken personal preferences of platforms into consideration: when not under time pressure, some readers who prefer the electronic platform actually show an effect of screen superiority^41^. In the current study, our focus with regard to the relationship between electronic device and reading will be on individual differences in the habits (and daily duration) of using electronic device, and the effect of these differences on reading comprehension.

## The Current Study

This study systematically investigates the relationships among executive functions, analogical reasoning, and electronic and non-electronic reading behaviour, and their impact on reading comprehension at both the behavioural and the neurocognitive levels. As previously mentioned, most neurocognitive studies of text comprehension have focused on narrative texts^28,42,43^. For example, the Extended Language Network hypothesis^42^ suggests that the classic language network, the semantic control and integration network, and the executive function network are simultaneously engaged during narrative text comprehension. Swett *et al*.^44^ was among the first to investigate the neural correlates of expository text comprehension. Consistent with the idea of multiple networks, Swett *et al*. reported patterns of co-activation in the brain’s key regions of cognitive control, visual processing, and language/semantic integration. Specifically, expository text comprehension also engages the core semantic-processing network for integrating word- and sentence-level semantic information, and additional multi-modal regions that create and update the situation/mental models for the text being read. The authors further reported different patterns for central versus peripheral text concepts, which implies that good readers notice and use the implicit *textual KS* of the expository text by focusing on the central and peripheral concepts differently (i.e., recruiting different regions of the brain).

In fMRI studies of reading, it is important to know the exact onset time of words and phrases to convolve the hemodynamic response function (HRF) with specific task-related variance and isolate it from unexplained variance. To this end, we employed a paradigm called “fixation-related fMRI”^45^ (see Methods for more details). Previous neuroimaging studies of texts dealt with the stimulus timing issue by controlling the presentation rate of the stimuli, typically with individual words, phrases, or sentences shown in a rapid-serial-visual-presentation or RSVP paradigm^46^. But reading every word for half a second in succession of one another is not a natural reading experience. To overcome this problem, we have taken advantage of an emerging paradigm that explores simultaneous eye-tracking and fMRI data acquisition (fixation-related fMRI). With this paradigm, participants are allowed to self-pace materials during reading in the scanner in a more naturalistic manner than reading via RSVP^47^. To match the fast speed of eye-movements and the cognitive processes during reading, we further used the multiband echo-planar imaging (EPI) acquisition technique^48^ to reduce the fMRI repetition time (TR) to 400 ms, in contrast to the typical TR of 2000 ms used in task-based fMRI studies. Multiband EPI provides greater within-participant statistical power with a higher sampling rate, a higher temporal Nyquist frequency to detect fast oscillatory neurally generated BOLD signals^49^, and better removal of spurious non-BOLD high frequency signal content^50^. By integrating eye-movement and high sampling-rate fMRI data in a naturalistic paradigm, our study is poised to provide neurocognitive insights into naturalistic scientific text comprehension.

To analyse the data collected from fixation-related fMRI, we incorporated a parametric modulator of the index of word position in sentences (starting from 1) in our fMRI GLM analysis. This approach aims to capture the variance in the HRF that changes along the time course of sentential processing across the text. It corresponds to the hypothesis of the Construction-Integration model^5,6^ that cycles of text comprehension roughly corresponds to short sentences or phrases^7,8^. Note that such a regressor, even though it is temporally based, would also capture variances associated with other concomitant cognitive processes which evolve along the time course of sentence reading (e.g., predictive pre-activation at syntactic, phonological, orthographic and perceptual levels^9^). Neural patterns negatively correlated with this regressor would be more involved in the early stage of sentential processing, which could be associated with the *construction* phase of the cycle or the integration of local information within the sentence. Neural patterns positively correlated with this regressor would be involved in the late stage of sentential processing, which could be associated with the *integration* phase, as well as the integration of the sentential information with more global context of the current textual representation or world knowledge. The beta images of this regressor (variance along the time course of reading a sentence) could be further used to investigate the effects of stimuli (e.g., textual KS) and individual differences (e.g., executive function). In a naturalistic reading paradigm such as used in the current study, these concomitant cognitive processes are not dissociable, and they are indeed vital in language comprehension^9^.

Given the approaches reviewed thus far, we make the following hypotheses. First, regarding the effects of *textual KS*, we hypothesize that when processing expository scientific texts with sub-optimal KS, cognitive demands of executive function should be higher due to the construction of a situation/mental model from the text; as a result, the associated neural correlates will be reflected as stronger activation in the executive control network, including the prefrontal cortex and the cingulate cortex. Second, regarding the effects of reader characteristics and individual differences, we hypothesize that executive function, analogical reasoning, and positive reading attitude will be positively correlated with reading comprehension performances. Neurocognitively, such correlations should be reflected as co-activation in areas including the left IFG, MFG, FPC, dorsolateral prefrontal cortex (DLPFC), and bilateral caudate heads, areas that are critical for executive function, analogical reasoning, and linguistic-semantic integration when processing scientific text^28,35,42–44^.

## Results

### Behavioural performances and individual differences

Participants read five expository texts in the scanner. Every participant made at least six correct answers to the 10 multiple-choice assessment questions at the end of each text during in-scanner reading. The accuracy for the questions for each text was as follows (mean% ± SD, n = 51): Mathematics, 94.71 ± 7.84, GPS, 90.98 ± 9.22, Mars, 91.76 ± 9.10, Electric Circuit, 95.10 ± 7.03, and Supertanker, 88.40 ± 11.14. ANOVA showed significant differences among participants’ performance accuracy on the texts (*F*_*(4*,*250)*_ = 5.32, *p* = 0.0004). Specifically, post-hoc Tukey’s HSD test showed that performance accuracy differed significantly between Electric Circuit and Supertanker (lower and upper confidence limit = 2.17, 11.94, *p* = 0.0009) and between Mathematics and Supertanker (LCL = 1.78, UCL = 11.55, *p* = 0.002).

Participants’ mean performance accuracy varied depending on the individual difference scores: it was positively correlated with GSRT scores (n = 49, *ρ* = 0.65, *p* < 0.0001), with Raven’s score of analogical reasoning (n = 49, *ρ* = 0.28, *p* = 0.027), and with reading preference index (n = 49, *ρ* = 0.27, *p* = 0.029). Further, the GSRT was also positively correlated with the working memory LNS task (n = 46, *ρ* = 0.36, *p* = 0.007) and with Raven’s scores (n = 49, *ρ* = 0.33, *p* = 0.011). GSRT scores also showed a positive trend though not significant correlation with the reading preference index (n = 49, *ρ* = 0.23, *p* = 0.059).

### fMRI Results: Main effects and individual differences of integrative processing

**Figure 2:**
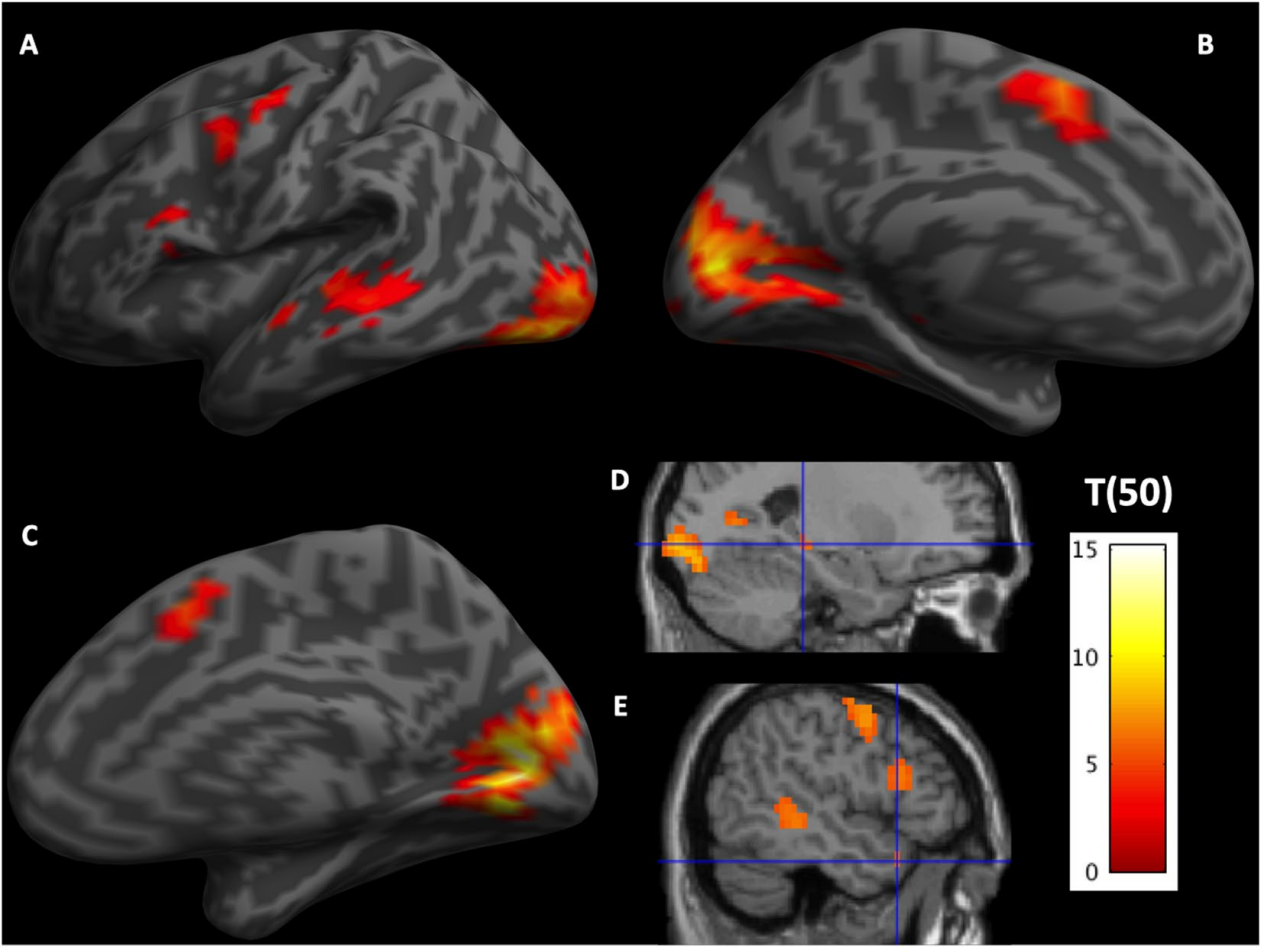
Neural correlates of content word processing. (**A**) Lateral view of the left hemisphere. (**B**) Medial view of the left hemisphere. Both showing significant voxels in the left visual cortex, SMA, precentral gyrus, IFG and STG and MTG. (**C**) Medial view of the right hemisphere showing the right visual cortex and SMA. (**D**) Sagittal section with cross hair at MNI [−24 −31 −6] highlighting the significant voxels in the left hippocampus. (**E**) Sagittal section with cross hair at MNI [−48 17 −26] highlighting the significant voxels in the left aTL.

Neural correlates of reading (Content Word fixation) were reflected in the strong activity in bilateral visual cortex and medial supplementary motor area (SMA), along with left precentral gyrus, superior and middle temporal gyrus (STG and MTG), anterior temporal lobe (aTL), inferior frontal gyrus (IFG) pars triangularis, and hippocampus (Table 1, Fig. 2).

**Table 1:** Neural Correlates of Reading and Integrative Processing.

H	Regions	Voxel	p	T	B.A.	[*x*, *y*, *z*]
Neural Correlates of Reading*						
B	Cuneus & lingual gyrus	1455	<0.001	15.16	23 & 18	12 −76 10
B	SMA	140	<0.001	9.04	6 & 32	−9 11 54
L	Precentral gyrus and MFG	112	<0.001	7.98	6	−42 −7 62
L	MTG & STG	179	<0.001	7.88	21	−57 −25 −2
L	IFG pars triangularis	30	0.001	6.5	45	−48 20 18
L	Hippocampus	8	0.002	6.25		−24 −31 −6
L	aTL	5	0.002	6.23	38	−48 17 −26
Neural Correlates of Early/Local Integrative Processing*						
R	IOG	49	<0.001	8.05		27 −91 −6
B	PCC & Precuneus	139	<0.001	7.82	31	−9 −52 26
L	Precentral	22	<0.001	7.64	6	−48 −4 42
R	VMPFC & pgACC	113	<0.001	7.4	10 & 32	9 53 −10
R	Cuneus	21	<0.001	6.92	17	12 −85 2
L	Fusiform, lingual & IOG	86	<0.001	6.74	19, 18 & 17	−24 −79 −18
B	pgACC	47	<0.001	6.72	32	−9 44 −2
L	IOG	9	0.012	5.72	19	−30 −88 14
R	Insula	5	0.012	5.7	13	42 −31 −6
Neural Correlates of Late/Global Integrative Processing*						
R	Lingual gyrus and cerebellum	212	<0.001	9.84	19	24 −61 −6
L	IPL & Supramarginal gyrus	126	<0.001	8.51	40	−39 −46 42
B	SMA	48	<0.001	8.14	32	−3 23 46
L	DLPFC & IFG pars triangularis	223	<0.001	8.12	6 & 46	−21 17 58
L	Insula	28	<0.001	7.82	13	−42 −7 10
L	Cuneus	49	<0.001	7.73	18	−6 −97 −6
L	Parahippocampal gyrus	44	<0.001	7.45	36	−30 −37 −14
L	MTG & ITG	50	<0.001	7.15	37 & 20	−57 −55 −10
R	DLPFC	38	<0.001	6.83	6	27 8 58
R	Precuneus	33	<0.001	6.74	31	27 −79 22
R	IFG pars triangularis	8	0.001	6.39	46	54 41 14
L	Precuneus & Angular gyrus	17	0.002	6.29	19	−30 −73 42
L	Cuneus	7	0.011	5.75	19	−9 −85 26
Neural Correlates of Integrative Processing for Individuals with lower E-device Usage**						
L	Insula & IFG pars triangularis	70	0.01	4.89	13 & 47	−30 17 14

^*^Voxel-level FWE corrected p-values.

**Cluster-level FWE corrected p-values with CDT p = 0.001 uncorrected.

Abbreviations: DLPFC = dorsolateral prefrontal cortex; IFG = inferior frontal gyrus; IOG = inferior occipital gyrus; IPL = inferior parietal lobule; ITG = inferior temporal gyrus; MTG = middle temporal gyrus; PCC = posterior cingulate cortex; SMA = supplementary motor area; VMPFC = ventromedial prefrontal cortex; H = hemisphere; L = left; R = right; p = p-value; T = T-value; B.A. = Brodmann area; *x*, *y*, *z* = MNI coordinates.

**Figure 3:**
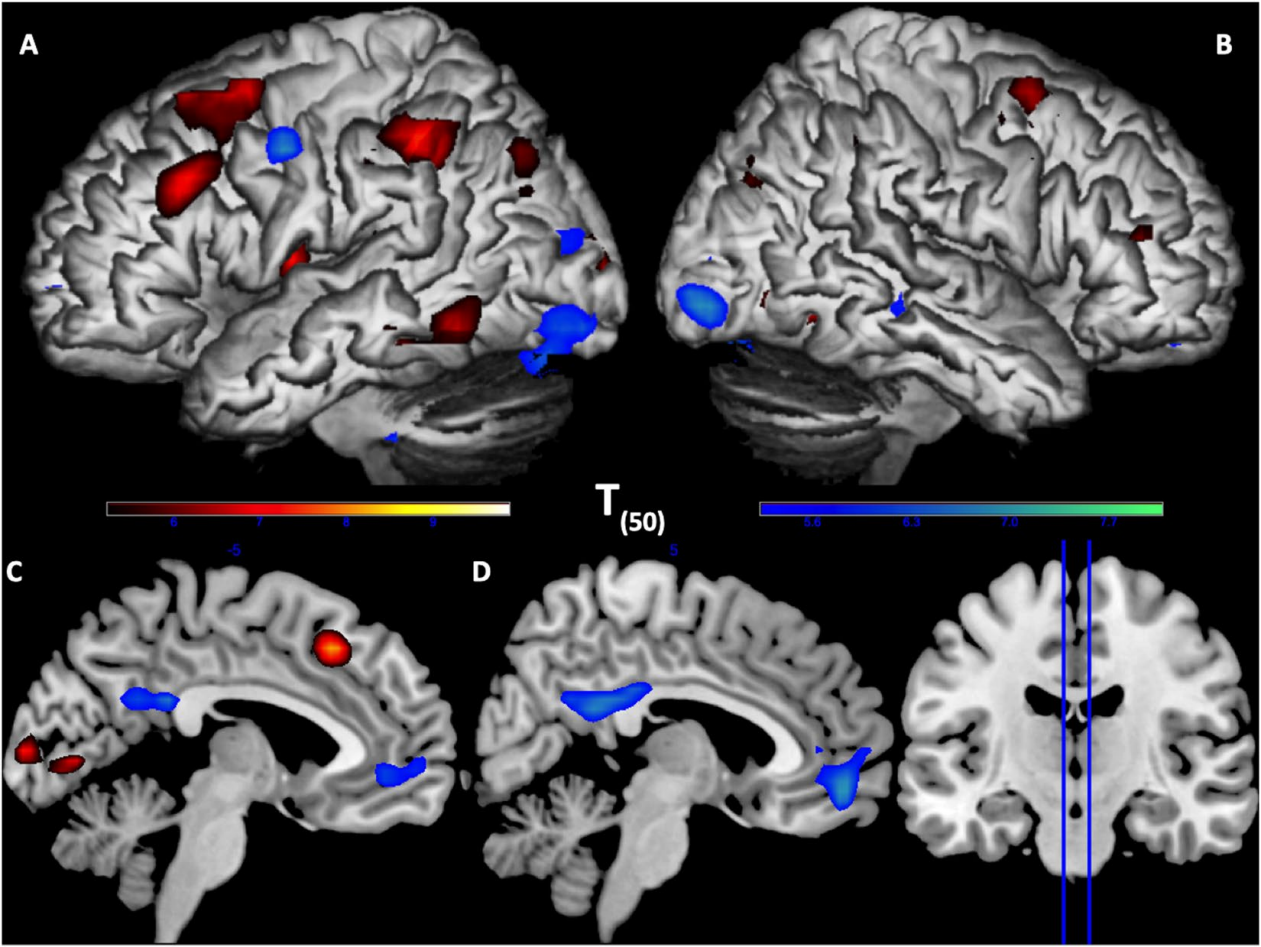
Neural correlates of integrative processing (word position effect). Surface rendering of negative (green) and positive (red) correlation with the word position index in sentences. (**A**) left hemisphere lateral view, (**B**) right hemisphere lateral view, (**C**) left hemisphere sagittal section of MNI *x* = −5, (**D**) right hemisphere sagittal section of MNI *x* = 5. Green regions are more activated in the beginning of sentences (negatively correlated with the word position index in sentences) including bilateral visual cortex, PCC and pgACC and left precentral gyrus. Red regions are more activated towards the end of sentences including bilateral DLPFC and IFG, left IPL, M&ITG and SMA.

**Figure 4:**
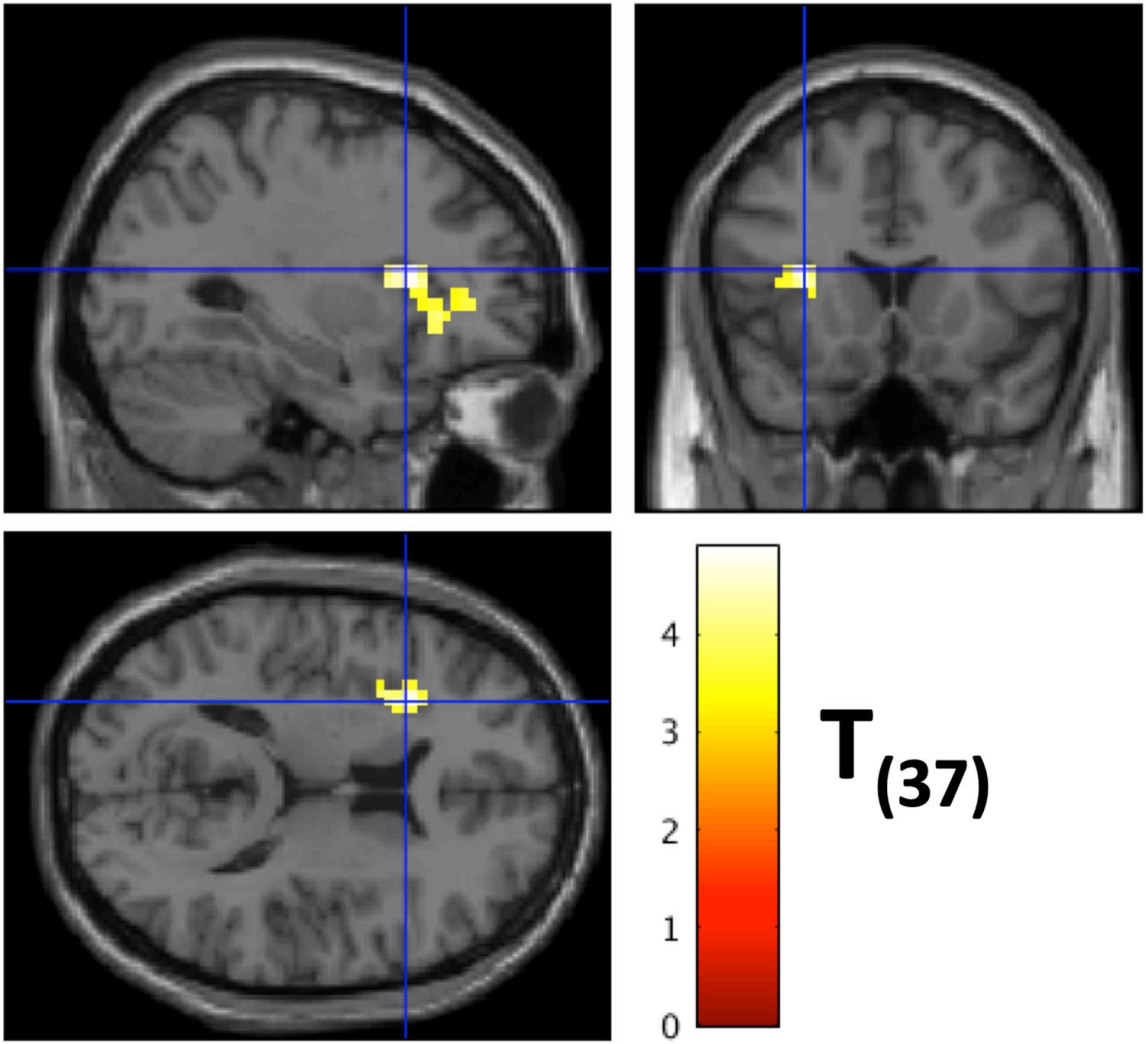
Neural correlates of integrative processing negatively correlated with individual E-device usage. The sections show the significant cluster in left insula and IFG pars triangularis in which the beta estimates for integrative processing were negatively correlated with the individual E-device usage reported in the RBQ. The crosshair highlights the peak in the cluster, MNI: [27 20 18].

Neural correlates of Integrative processing were reflected in two different patterns: the first one, negatively correlated with the word position regressor, was associated with strong activities in bilateral occipital pole, posterior cingulate cortex (PCC), pregenual anterior cingulate cortex (pgACC), as well as left fusiform and precentral gyrus (Table 1, Fig. 3, blue); the second, positively correlated with the word position regressor engaged DLPFC, IFG pars triangularis, precuneus, lingual gyrus, MTG and ITG, as well as left IPL, medial SMA, insula, and the parahippocampal gyrus (PHG) (Table 1, Fig. 3, red). One cluster in the left insula and IFG pars triangularis showed negative correlation between the E-device reading index and Integrative processing (MNI: [27 20 18]; Table 1, Fig. 4).

### fMRI Results: Main effects and individual differences of KS optimality

**Figure 5:**
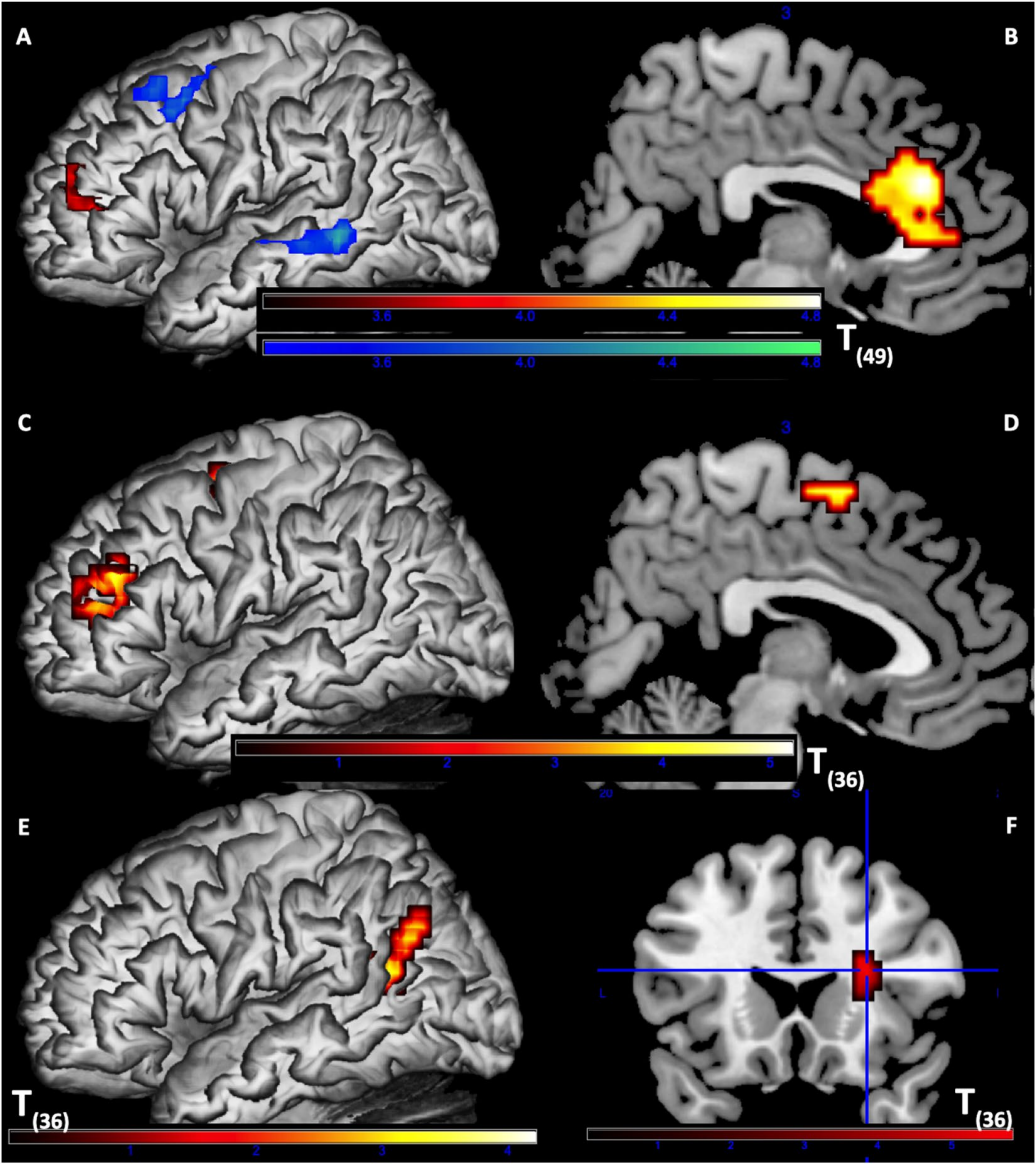
Neural correlates of KS optimality and individual differences. **(A**,**B**) Neural correlates of integrative processing for texts with different KS optimality. Red: neural correlates of texts with sub-optimal KS (quadratic MBC values away from 0), including bilateral dACC and left FPC and IFG pars triangularis. Green: neural correlates of texts with optimal KS (quadratic MBC values closer to 0), including left DLFPC, STG and MTG. Panel B showed the sagittal section of MNI *x* = 3. (**C**,**D**) Neural correlates of texts with sub-optimal KS in individuals with higher GSRT scores, including left FPC and SMA. Panel D showed the sagittal section of MNI *x* = 3. (**E**) Left IPL correlated with sub-optimal KS processing in individuals with higher E-device usage. (**F**) Right claustrum (crosshair MNI = [27 20 18]) correlated with sub-optimal KS processing in individuals with lower E-device usage.

After the linearly correlated variance of MBC (maximum betweenness centrality) was partialled out, the quadratic term of MBC represented the optimality of *textual KS* (with values closer to 0 being more optimal; see *Introduction*). Neural correlates of the processing of texts with optimal KS revealed strong activity in the left DLPFC and left middle STG, while the processing of sub-optimal KS led to greater activity in the left frontopolar cortex (FPC) and bilateral dorsal ACC (Table 2, Fig. 5A,B). Furthermore, left FPC and bilateral SMA were correlated with the processing of sub-optimal KS texts among participants with higher GSRT scores (Fig. 5C,D), suggesting an interaction between textual KS properties and reader characteristics (e.g., of high-vs-low reading competence). Finally, this text-reader interaction was also reflected in the regression results of E-device reading index: during processing of sub-optimal KS texts, neural responses in the left temporoparietal junction (TPJ, Fig. 5E) increased with E-device reading index, while responses in the right claustrum (Fig. 5F) decreased. These interactions have significant implications for student science concept learning, as discussed below.

**Table 2:** Neural Correlates of Optimality of KS.

H	Regions	Voxel	p	T	B.A.	[*x*, *y*, *z*]
***Neural Correlates of Integrative Processing for Texts with Optimal KS***						
L	MTG & STG	122	0.001	4.81	22 & 21	−51 −46 −2
L	DLPFC (SFG & MFG)	92	0.003	4.66	6 & 8	−48 11 50
***Neural Correlates of Integrative Processing for Texts with Sub-optimal KS***						
B	dACC	204	<0.001	5.21	32	3 35 26
L	FPC (MFG) & IFG pars triangularis	66	0.015	4.42	46 & 10	−39 44 10
***Neural Correlates of Sub-optimal KS Processing in Individuals with higher GSRT***						
L	FPC (MFG)	77	0.007	5.19	10	−30 38 26
L	SMA	70	0.01	5.09	6	−12 −1 62
***Neural Correlates of Sub-optimal KS Processing in Individuals with higher E-device Usage***						
L	TPJ (MTG & Angular gyrus)	50	0.039	4.21	22 & 39	−36 −58 18
***Neural Correlates of Sub-optimal KS Processing in Individuals with lower E-device Usage***						
R	Claustrum	53	0.031	5.81		27 20 18

*Cluster-level FWE corrected p-values with CDT p = 0.001 uncorrected.

Abbreviations: dACC = dorsal anterior cingulate cortex; DLPFC = dorsolateral prefrontal cortex; FPC = frontopolar cortex; IFG = inferior frontal gyrus; MFG = middle frontal gyrus; MTG = middle temporal gyrus; SMA = supplementary motor area; STG = superior temporal gyrus; TPJ = temporoparietal junction; H = hemisphere; L = left; R = right; p = p-value; T = T-value; B.A. = Brodmann area; *x*, *y*, *z* = MNI coordinates.

## Discussion

The current study investigated the neurocognitive processes underlying the interaction between properties of expository texts and characteristics of the reader, specifically between the *textual KS* (network structure of the texts to be read) and the individual readers’ executive function, reasoning, and reading habits. Our study also showed that readers’ electronic device usage is negatively correlated with the involvement of key brain regions for integrative information processing. To our knowledge, this study is the first systematic behavioural and neurocognitive investigation of expository texts of scientific concepts with a naturalistic reading paradigm that combines both fMRI and eye-tracking.

First, at the behavioural level, we found that student performance in reading comprehension is correlated with individual differences in executive functions, analogical reasoning, and positive reading attitude. The GSRT general reading ability scores are correlated with analogical reasoning and positive reading attitude, for both in-scanner performance and immediate post-test assessment questions. GSRT scores were also correlated with individual differences in working memory. These patterns are in line with previous studies that have identified the relationships between reading comprehension and executive functions^30^ and between comprehension and reading behaviour^38^.

The relationships among reading comprehension and executive function, reasoning, and reading attitudes are not one-to-one, but are multidirectional and complex. For example, better executive function might lead to superior reading comprehension, and conversely, better reading experience could improve readers’ reasoning, attention, and working memory. Readers with a positive reading attitude engage in more reading activity, which leads to more rewarding experiences and in turn more positive reading attitude. Different reader characteristics could also be related to each other: for example, reasoning has been proposed to require working memory capacity in the mental model theory^15^, engaging working memory’s underlying executive processes^51^. Finally, reading comprehension performance may be correlated with the student’s success in other domain disciplines: reasoning abilities have been found to be predictive of academic achievements in Mathematics, Biology, Physics, History, and English^52,53^. Our findings that scientific text reading comprehension is correlated with individual differences in working memory and analogical reasoning are consistent with these general findings but also more specifically demonstrate that individual difference variables impact scientific reading. Although these correlations could have underlying causal relations, the current study was not designed to test causal relationships, which need to be investigated in future studies.

Second, at the neurocognitive level, we found dynamic neural correlates of integrative information processing, suggesting a local predictive focus on surface form analysis (visual cortex and fusiform gyrus) and a global predictive focus on semantic, syntactic analysis and integration (frontoparietal network and SMA). Such change in focus across the time course of processing is in line with the different time-scale analysis of text comprehension. At the beginning of reading a new sentence, integration of local information within the sentence takes place, which demanded primary perceptual-motor areas with short process memory. The more the reader proceeds along the sentence, the more the integration of sentential information with global context of the current textual representation or world knowledge takes place, which demanded higher order areas with long process memory, as predicted by models of memory and text comprehension^6,9–11^. Thus, temporal ordering and integrative processing may be related at multiple levels and time scales, although the predictive pre-activation hypothesis^9^ emphasizes that integrative processing is due to parallel integration rather than staged processing across time.

With regard to the impact of text properties, texts that have optimal *textual KS* recruit regions associated with linguistic, semantic (IFG and temporal lobe), and integrative processing (DLPFC). Texts with sub-optimal *textual KS* recruit regions that are critical for dual-tasking, monitoring, and attention (FPC and dACC), suggesting that these texts elicit more effortful processing during mental model construction. Furthermore, reading competence (as measured by GSRT scores) is reflected clearly in the processing of texts with sub-optimal KS: high-competence readers activate regions in integrative information processing in the SMA and FPC, as well as regions for linguistic processing in IFG, insula and STG, suggesting the engagement of multiple brain networks for conceptual integration.

Due to the nature of the hemodynamic response function, we used the content word fixation regressor to capture the variance of neural responses throughout text reading. Neural correlates of this regressor included the typical fronto-temporal circuit engaged in language, syntactic and semantic processing (IFG, STG, MTG, aTL)^42,54^, but also the SMA and hippocampus. SMA, including the supplementary eye field (SEF) and the pre-SMA which has traditionally been implicated in motor planning and motor learning^55^. However, in the context of semantic retrieval, Danelli *et al*.^56^ found the SMA, premotor, and left IFG to be involved in both grapheme-to-phoneme and lexical-semantic routes of lexical access. Further, pre-SMA has been proposed to be part of a network including thalamus and caudate nucleus that govern aspects of semantic retrieval of object memories, supported by EEG data^57^. The left SMA is also associated with syntactic processing as shown in a recent meta-analysis^54^. The SMA and pre-SMA activity could be part of the on going predictive pre-activation process across multiple levels during reading comprehension^9^. In addition, Duff and Brown-Schmidt^58^ proposed that the hippocampal declarative memory system is a critical contributor to language use and processing because of its capacity for relational binding, representational integration, flexibility, and maintenance. In Hasson *et al*.’s memory processing hierarchy^10^, the medial temporal hippocampal region would also interact with regions with long process memory, and facilitate binding and consolidation of incoming information with global context and world knowledge. Given these findings in the literature, it is not surprising that SMA and the hippocampus both play crucial roles in expository text comprehension as shown in our current study, since the predictive and integrative processes take place irrespective of the text genre (i.e., narrative or expository).

Augmented by the high-sampling rate (400 ms TR, a Nyquist frequency of 800 ms) of multiband EPI acquisition in our current design (see Methods), the parametric modulator of word position in sentences successfully captured the dynamic change of neurocognitive integrative processes along different time scales during reading comprehension (mean reading time for each sentence = 3.33 ± 0.86 s). Our results indicated that the temporal evolution of integrative processes shifted from relatively shallow, form-oriented and local processing (e.g., involving the occipital cortex and fusiform gyrus) to more global processing that involves semantic retrieval, information integration, and situational/mental model updating that engage the DLPFC, IFG, IPL, and SMA. Previous work based on narrative text reading has implicated the frontoparietal network in situation model building, an integrative mental representation of the text, with a rough division of labour in situation model construction (the posterior parietal and anterior temporal regions) and situation model maintenance (frontal regions)^46^. Our finding of the dynamic changes at the sentential level, although from scientific rather than narrative text reading, is consistent with the theoretical framework that the situation/mental model is constantly updated as reading comprehension unfolds in time^14,59^. Such dynamic changes are seen in cognitive domains other than language or reading: for example, Fangmeier *et al*.^60^ showed a similar pattern of shift in neural correlates during different stages of reasoning in which the initial processing of the premise involves occipital and temporal regions, whereas the validation of a given conclusion based on the premise engages the frontoparietal network (DLPFC, IPL, and precuneus).

By modelling the knowledge structure of a text as network maps (e.g., *textual KS*), we were able to capture the differences in the neural correlates of expository science text reading as a function of text structure. Specifically, the graph-theoretical measure MBC (referred to as graph centrality) of a *textual KS* network allowed us to represent texts with optimal (network-like maps) vs. sub-optimal (spoke- or chain-like) KS^18^, and such KS differences directly impact the neurocognitive substrates of reading. Previous behavioural studies^22–25^ have suggested an inverted U-shape function between network graph centrality of knowledge structure and reading comprehension performances. By using the U-shaped quadratic term of knowledge structure as regressor, we found that the processing of optimal KS texts recruits classical language processing brain regions (left M/STG), along with regions that involve situation/mental model construction and information integration (left DLPFC), whereas processing of sub-optimal KS texts engaged activities in the left FPC and bilateral dorsal ACC.

In the context of multitasking research, FPC and ACC have been proposed to serve complementary but dissociable roles in allocating resources for cognitive control of the primary and subgoals/tasks^61,62^. While ACC has been frequently implicated in language processing (especially conflict monitoring in bilingual speech production)^63^, the role of FPC (Brodmann Area 10) has been traditionally linked to a variety of higher-order cognitive functions based on human and primate research^64^. Specifically, FPC has been associated with the ability to hold a primary goal while performing concurrent subgoals, playing an important role in multitasking and multiple resource allocation^61,65–67^, including reasoning and integration of multiple disparate mental relations^68^. Given this role of FPC in integrative processing, it is no surprise that we see it involved in the processing of sub-optimal KS texts that have (1) spoke-like KS, where a core concept is associated with multiple isolated concepts, and (2) chain-like KS, where concepts are serially associated one by one. In these cases, multitasking is required of the reader so as to retain the core concept while processing and integrating multiple isolated sub-concepts across the text. Note that the quadratic effect of graph centrality (as measured with MBC) in FPC and ACC in our data cannot be accounted for by its relation with other psycholinguistic variables such as word length or word frequency, although the latter have also been shown to have curvilinear/quadratic effect on both behavioural^69^ and neuroimaging correlates of reading^70^. It is important to note that MBC measures of the texts are largely collinear with the text-wise mean values of key psycholinguistic variables such as word frequency, AoA, and word length (see Section *Materials* in Method). However, in our subject-level regression model, we included both linear and quadratic terms of MBC, and the linear term was included as a covariate of non-interest. Therefore, the confounding linear effects of the psycholinguistic variables were partialled out before the group-level multiple regression.

The impact of electronic device usage is evident in our results. Across all texts, we found a negative correlation between frequency in electronic device usage and BOLD activity in left insula and IFG pars triangularis. The anterior insula is part of the salience network^71^, which responds to the degree of information saliency (and subsequent attention) in a variety of domains including cognitive and emotional processing^72–74^. Sridharan *et al*.^75^ used Grainger Causality to estimate effective connectivity, proposing that the fronto-insular cortex plays a critical and causal role in switching between the central-executive network and the default-mode network. In addition, our data indicate that individuals with higher electronic device usage, on the one hand, have decreased engagement in insula and IFG, and on the other, recruit more left TPJ and less right claustrum when processing texts with sub-optimal KS. The claustrum has the highest connectivity in the brain by regional volume, especially with the frontal lobe and cingulate regions^76^, and it has been proposed to be the “gate keeper” of neural information for conscious awareness^77^. Considering the potential negative effects of excessive daily usage of electronic device (especially texting on smartphones), the neural patterns in our data regarding insula and claustrum, along with the behavioural data of Follmer *et al*.^38^, could point to the readers’ reduced or inefficient coordination of cognitive resources and switching between the central executive and default mode networks. At the same time, the result of over-engagement of the TPJ, part of the executive network^71^, might suggest that these same readers required more effortful processing, especially for texts with sub-optimal KS of the spoke or chain types.

Finally, we found that individuals with higher GSRT scores engage the left FPC and bilateral SMA more strongly when reading texts with sub-optimal KS. As discussed above, FPC and SMA may be significant for expository text comprehension given their important roles in multi-tasking, cognitive resource allocation, and visuospatial processing. Our neurocognitive patterns suggest that better reading ability is associated with the engagement of neural substrates responsible for highly integrative cognitive processes as well as for reasoning. By contrast, readers who report excessive daily electronic device usage may not activate these critical brain regions for integrative cognitive processing. As discussed in the *Introduction*, behavioural work on the immediate effect of media (paper vs. screen) has, by and large, shown that excessive use of screen-based devices is associated with lower quality of metacognitive processes^40,41^. Our findings provided the first neurocognitive evidence that habitual electronic device usage might adversely affect high-level cognitive processing required for scientific text comprehension. Future investigation is needed to identify the causal relationships among reading habits, preferences of media types, metacognitive performances, and expository text comprehension.

## Methods

### Participants

Sixty-two right-handed native English speakers were recruited. Seven participants did not finish the first session due to eye-tracker or MR scanner technical issues. One participant was excluded due to very low accuracy (50%) for an in-scanner comprehension test and poor behavioural testing results outside the scanner. One participant was found to be left-handed after the behavioural session, leaving 51 participants aged between 18 and 40 years in our analysis. Eye-tracking data were missing for one participant during one run containing one text, leading to its exclusion for the analysis for KS. Forty-nine out of the 51 participants completed the behavioural testing session, of which only 46 correctly performed the Letter Number Sequencing task. Therefore, behavioural data analysis included 49 participants (23 males, mean age ± SD = 22.69 ± 4.57). fMRI data for neural correlates of Reading and Integrative Processing included 51 participants (24 males, mean age ± SD = 22.67 ± 4.52). Forty-six participants (21 males, mean age ± SD = 22.84 ± 4.63) were included in the fMRI multiple regression models for neural correlates of individual differences in Integrative Processing. Forty-five participants (21 males, mean age ± SD = 22.47 ± 3.88) were included in fMRI regression models for neural correlates of individual differences in sentential processing of texts with different KS optimality.

All participants had normal or corrected to normal vision, and had no history of mental or neurological disorder. The study was approved by the Pennsylvania State University Institutional Review Board (IRB) and was performed in accordance with the ethical standards described in the IRB. Written informed consent was obtained from all participants before they took part in the study.

### Materials

Prior to the experiment, five expository texts of STEM contents were modified from previous research stimuli (see Follmer^38^ for details): Mathematics (Permutations and Combinations, 28 sentences, 306 words, maximal betweenness centrality/MBC = 0.34), GPS (28 sentences, 307 words, MBC = 0.29), Mars (31 sentences, 310 words, MBC = 0.59), Electric Circuit (30 sentences, 302 words, MBC = 0.16), and Supertanker (31 sentences, 302 words, MBC = 0.72). Texts were controlled for the mean word count per sentence (10.4 ± 0.62) and the mean character count per sentence including spaces (62.48 ± 1.92). Furthermore, psycholinguistic variables of the lexical properties (word frequency, length, etc.) of each text were derived from the English Lexicon Project^78^ the Kuperman age-of-acquisition (AoA) database^79^, the MRC Database^80^ and the Brysbaert concreteness database^81^. Bootstrapped One-way ANOVAs revealed no significant difference between the average values across all five texts for the average number of syllables (NSyll, *F* = 0.05, *p* = 0.99), lexical decision time (LDT, *F* = 1.07, *p* = 0.38), log frequency (*F* = 0.25, *p* = 0.91), naming response time (NRT, *F* = 1.41, *p* = 0.23), orthographic neighbourhood density (OLD, *F* = 0.04, *p* = 0.99), phonological neighbourhood density (PLD, *F* = 0.34, *p* = 0.85), concreteness (*F* = 0.24, *p* = 0.91), and number of phonemes (NPhon, *F* = 0.02, *p* = 0.99). However, one-way ANOVAs for average word length and AoA were significant at p < 0.05 (*F* = 3.27, *F* = 3.32, respectively). Text-wise, mean values of psycholinguistic variables were linearly correlated with the linear term of MBC (OLD, *r* = 0.92 *p* = 0.025; PLD, *r* = 0.92, *p* = 0.0285; NSyll, *r* = 0.92, *p* = 0.0268; NPhon, *r* = 0.88, *p* = 0.0487; LDT, *r* = 0.9, *p* = 0.0356; NRT, *r* = 0.91, *p* = 0.034; frequency, *r* = 0.89, *p* = 0.0453; AoA, *r* = 0.88, *p* = 0.049; length, *r* = 0.87, *p* = 0.053, concreteness, *r* = 0.88, *p* = 0.0487). Stimuli were presented using E-Prime 2.0^82^, sentence by sentence onto a screen which was then projected onto a reflective mirror mounted above the participants’ eyes in the MRI scanner (see section *Eye-tracking Data Acquisition and Processing* for details).

### KS quantified as maximal betweenness centrality (MBC, Graph Centrality)

Fifteen key terms were selected as nodes from each of the five texts^38^, along with their synonyms and metonyms. The key terms were aggregated from a key-term generating task of a previous Amazon MTurk study of 403 participants^38^ and a key-term generating task of the authors of the current study (with a general overlap of 88%). The edges between the nodes are defined as proximity associations between nodes, operationalized as follows: a forward pass is made through the text without regard to sentence boundaries, and for every key term that is found, it is linked to the immediate previous key term by entering a “1” (binary coding) in a 15 by 15 term proximity array, indicating that there is a link (edge) between the two terms. Textual network maps were thus generated with *Analysis of Lexical Aggregates Reader (ALA-Reader)*^19^. Maximal Betweenness Centrality per map/text as a measure of graph centrality (and measure of KS) was calculated using the NodeXL software (Microsoft Inc., 2018). For a node *k* in a network, its partial betweenness with respect to the other two nodes *i* and *j* is defined as the probability that node *k* falls on a randomly selected path linking nodes *i* and *j*. The betweenness centrality value of node *k* is the sum of the partial betweenness values in respect to all pairs of nodes in the network except for *k*^27^. Each node in a network has a betweenness centrality value. Note that the betweenness centrality measure depends on the number of nodes in the graph^27^, and the absolute value of MBC per se does not indicate the optimality of KS. In the current study, the lengths of all the texts were made comparable (roughly 300 words), and we used 15 nodes (key concepts) to construct concept maps for all five texts so that the graph centrality values and the range of optimal KS values are also comparable across the texts. To operationalise the optimality of *textual KS*, the centrality values were normalised and quadratic terms were calculated. Higher quadratic centrality values (further away from zero, which is the average in the normalised distribution) indicate sub-optimal KS, while lower quadratic centrality values (closer to zero) indicate more optimal KS.

### Procedure

After providing consent, participants underwent a structural MRI scan, followed by a practice session for self-paced reading in the scanner. They were instructed to click a button to advance from one sentence to the next. Each sentence was presented for up to 8 seconds after which the next sentence automatically appeared on the screen. At the end of each text they answered 10 comprehension questions. Once the practice session ended, the participants completed five self-paced reading sessions, during which time simultaneous fMRI and eye-tracking data were collected. On a second visit, which was usually one week after the in-scanner reading session, participants completed a battery of behavioural tests.

### Behavioural data collection and processing

In the behavioural session, the Gray Silent Reading Test, Raven’s Progressive Matrices, Letter Number Sequencing and Attention Network tests were presented to participants via E-Prime 2.0, and the Reading Background Questionnaire was completed on an internet browser. Detailed information of each test is as below.

#### Gray Silent Reading Test (GSRT)

The GSRT test measures reading comprehension competence^83^. Up to 13 narrative texts were provided the in GSRT, and each text was presented alongside five assessment questions. Adult participants started with Text No. 8 (a text of middle-level difficulty) and were tested downward (e.g., Text No. 7) until the basal was reached (i.e., when all five questions were answered correctly), and upward (e.g., Text No. 9) until the ceiling was reached (i.e., 3 out of 5 answers were wrong). Because all participants were in the same age group (18 and beyond), conversion of scores to quotient according to age groups was not necessary, and the raw scores were used.

#### Raven’s progressive matrices

The Raven’s test measures analogical reasoning^34^. In each of the sixty-five tests, a matrix of relations, from which part is omitted, is presented. Subjects have to choose, from a group of six or eight alternatives, the one which completes the matrix. The problems are arranged in five sets, each of which has a distinctive theme: (A) continuous patterns, (B) analogies between pairs of figures, (C) progressive alterations of patterns, (D) permutations of figures and (E) resolution of figures into constituent parts. The first problem in a set is intended to be self-evident, and it is succeeded by twelve problems of increasing difficulty. The testing time was limited to 10 minutes, and the number of corrected trials was used as the score.

#### Letter number sequencing (LNS)

The LNS task measures working memory. The task was adapted from the Wechsler Adult Intelligence Scale (WAIS-III)^33^. Participants heard a series of alternating letters and numbers and were asked to recall the numbers first in ascending order and then the letters in alphabetical order. The task began with a set size of two (one letter plus one number) and increased by one for every three trials until a set size of eight was reached. The participants’ outputs were corrected for using capital letters (if lower-case letters were the targets) and accidental usage of arrow keys. To properly reflect the difficulty of different items, size-weighted scores were calculated as the summation of correct items’ set size. For example, if the participant was correct in three items with the size of two, one item with the size of three, and two items with the size of four, the score will be calculated as 3 × 2 + 1 × 3 + 2 × 4.

#### Attention network test (ANT)

The ANT tests measure the alerting and orienting skills of attention and the inhibitory control ability of executive function^32^. It consisted of a flanker test in which a central arrow was presented with congruent or incongruent flanking arrows, and the participants were asked to give indicate the direction of the central arrow as fast and as accurately as possible. The row of arrows could appear above or below the fixation cross. In some trials before the arrows appeared, one or two asterisks would appear. They could either alert the participants that the arrows will appear soon but without orienting the location of the arrows, or alert them that the arrows will appear soon and direct attention to the correct location (orienting). Three scores were derived according to Fan *et al*.^32^, reflecting the RT differences caused by alerting, orienting, and conflicting manipulations; for example, the higher the conflict effect on RT, the lower the participant’s inhibitory control is.

#### Reading Background Questionnaire (RBQ)

Participants were administered 20 questions constructed based on previous research^84,85^ to assess readers’ general reading habits and background, using a Google Form^38^. The items asked about participants’ reading habits on electronic media (e.g., computers, smartphones), their electronic non-reading behaviour (e.g., time spent texting friends, watching television), and their reading habits (amount of time spent on reading), preferences (e.g., enjoyment of types of books, enjoyment of books about different cultures), attitudes towards reading, and reading ability. Items were administered using either a 4-point or a 5-point Likert scale.

Correlational analyses showed significant correlations between E-device reading and non-reading time, and pair-wise correlations among reading time, reading preference and reading attitude/ability (see also Follmer *et al*.’s analyses^38^ of how these variables impact reading). Exploratory factor analysis yielded two factors: Factor 1 explains 34.45% of variance, including reading preference (loading = 0.90), reading attitude/ability (loading = 0.66) and reading time (loading = 0.64); Factor 2 explains 21.67% of variance, including E-device reading (loading = 0.97) and non-reading time (loading = 0.31). Given these two factors, we simplified the RBQ variables into two scores: E-device reading index (summation of E-device reading and non-reading time) and reading preference index (summation of reading preference, reading attitude/ability and reading time).

### Behavioural data analyses

To test what cognitive measures contribute to participants’ reading comprehension behaviourally, we performed non-parametric correlation tests checking correlations between GSRT or question-answering accuracy with the Raven’s scores, LNS scores, the ANT Alerting, Orienting, and Conflict scores, the RBQ E-device reading and reading preference indices. Because the mean accuracy of the performance assessment scores and the GSRT scores violated the assumption of normality (Shapiro-Wilk W test, both *p*s < 0.01), one-tailed non-parametric Spearman’s correlations were used.

### Eye-tracking data acquisition and processing

The basic idea of fixation-related fMRI paradigm, as first explored by Marsman *et al*.^86^, is to use self-paced eye-movements to convolve the hemodynamic responses and model the psychological regressors to analyse fMRI data of visual processing. Later studies^45,87^ have further demonstrated the validity of simultaneous eye-tracking and fMRI paradigms in naturalistic word and text reading. Eye movements were recorded with an Eye-Link 1000 Plus long-range mount MRI eye tracker (SR-Research) with a sampling rate of 1 kHz. The camera was placed at the rear end of the scanner bore, and captured eye movements via a reflective mirror above the head coil. The distance between the camera and the participant’s eyes via the reflective mirror was 120 cm. Recording was monocular (from the right eye), and the participant’s head was stabilized in the head coil. A 13-point calibration routine preceded the experiment. Before each reading session, a validation procedure is performed, and re-calibration is done when the validation error is larger than 1 degree.

Data adjustment was later performed to address drifting issues caused by the calibration accuracy decline over time. For fixations falling outside (above or below) the range of predefined target regions, manual adjustment was performed using the Data Viewer software. Instead of using auto-adjustment which brings all fixations onto one horizontal line, we performed trial-by-trial correction adjusting all of the fixations in a single try only along the y axis (vertical adjustment) so as to maintain readers’ original eye fixation patterns.

### MRI data acquisition

Data were acquired using a 3 T Siemens Magnetom Prisma Fit scanner with a 64-channel phased array coil. We acquired a MPRAGE scan with T_1_ weighted contrast [176 ascending sagittal slices with A/P phase encoding direction; voxel size = 1 mm isotropic; FOV = 256 mm; TR = 1540 ms; TE = 2.34 ms; acquisition time = 216 s; flip angle = 9°; GRAPPA in-plane acceleration factor = 2; brain coverage is complete for cerebrum, cerebellum and brain stem]. After the T_1_, we acquired five functional runs of T_2_* weighted echo planar sequence images [30 interleaved axial slices with A/P phase encoding direction; voxel size = 3 × 3 × 4 mm; FOV = 240 mm; TR = 400 ms; TE = 30 ms; acquisition time varied on the speed of self-paced reading, maximal 306 s; multiband acceleration factor for parallel slice acquisition = 6; flip angle = 35°; brain coverage misses the top of the parietal lobe and the lower end of the cerebellum]. Additionally, we collected a pair of spin echo sequence images with A/P and P/A phase encoding direction [30 axial interleaved slices; voxel size = 3 × 3 × 4 mm; FOV = 240 mm; TR = 3000 ms; TE = 51.2 ms; flip angle = 90°] to calculate distortion correction for the multiband sequences^88^.

### fMRI data preprocessing and analyses

Data preprocessing and analysis were performed in SPM12 v6906 (http://www.fil.ion.ucl.ac.uk/spm). Functional imaging preprocessing consisted of correction of field inhomogeneity artefacts with the HySCO toolbox (Hyperelastic Susceptibility Artifact Correction)^89^ using the pair of spin echo sequence images and realignment for motion correction. The structural image was coregistered to the mean functional image, and segmented into grey matter, white matter, cerebrospinal fluid, bone, soft tissue, and air/background to estimate the forward deformation parameters to MNI space. Images were normalized with the 4^th^ degree B-Spline Interpolation algorithm and further smoothed with a Gaussian kernel of 8 mm full-width-at-half-maximum (FWHM).

In the GLM analysis, the design matrix contained one psychological regressor of interest, the “Content Word” condition, specifying the onsets and gaze durations of first pass fixations and regressions for content words (informed by eye-tracking data). The index of word position in sentences (starting from 1) was incorporated as a parametric modulator of the “Content Word” condition. We also included two psychological regressors of non-interest: “Non-Content Word” and “Instructions”: the “Non-Content Word” condition modelled fixations on non-content (function) words and ocular regressions, and the “Instructions” condition modelled two seconds of instructions presented at the beginning of each run. Because of the self-paced reading, all psychological regressors at the first level were subject-specific. Finally, we included six motion parameters and three physiological regressors (white matter, ventricular, and non-ventricular CSF space signal). We then applied a high pass filter with a cut off period of 128 s, and the temporal autocorrelation was accounted for with the FAST option in SPM12^90^. Then, we calculated fixed effects across all runs for each subject. At the group level, two random-effect one sample t-tests (N = 51) were performed for the effects of reading in general (Content Word fixation), and Integrative Processing (parametric effect of word positions). We applied peak-level family-wise error (FWE) correction of p < 0.05, minimal cluster size = 5 voxels, for the main effects of both one sample t-tests.

At the group-level, the beta maps of the Integrative Processing obtained at the subject-level were entered into one multiple regression model as the dependent variable (N = 46). The following eight independent variables were included to checked the effect of individual differences: (1) GSRT, (2) Raven’s, (3) span-weighted LNS, the (4) Alerting, (5) Orienting, and (6) Conflict effects of the ANT, (7) the RBQ E-device reading index and (8) the RBQ reading preference index. At the whole brain level, we applied cluster-level FWE-correction p < 0.05, using a cluster-defining threshold of p = 0.001.

To further investigate Integrative Processing due to the effects of *textual KS* (measured as MBC, see *Materials* in the Methods), the beta maps of Integrative Processing of each text were entered into a subject-level regression model including the linear and quadratic terms of MBC as the independent variable. At the group level, the beta maps of quadratic MBC correlates of the Integrative processes were entered into an one-sample t-test (N = 50) for the main effect and a multiple regression model (N = 45) with the same eight independent variables for individual differences as mentioned before. We applied cluster-level FWE-correction p < 0.05, using a cluster-defining threshold of p = 0.001, for the main effects of the one-sample t-test and for each individual difference in the multiple regression model of MBC.

## Supplementary information

Supplementary Information

## Data Availability

All behavioural, eye-tracking and neuroimaging data (with personal information de-identified) have been made available on OpenNeuro (https://openneuro.org/datasets/ds001980/versions/1.0.1) and on the PI’s lab website (http://blclab.org/).
