# Author Correction: Neurocognitive Signatures of Naturalistic Reading of Scientific Texts: A Fixation-Related fMRI Study

**DOI:** 10.1038/s41598-020-68554-6

**Published:** 2020-07-07

**Authors:** Chun-Ting Hsu, Roy Clariana, Benjamin Schloss, Ping Li

**Affiliations:** 1 Department of Psychology and Center for Brain, Behavior, and Cognition, Pennsylvania State University, University Park, PA 16802 USA; 2 Kokoro Research Center, Kyoto University, 46 Yoshidashimoadachi-cho, Sakyo-ku, Kyoto, 606-8501 Japan; 3 Department of Learning and Performance Systems, Pennsylvania State University, University Park, PA 16802 USA

Correction to: *Scientific Reports* 10.1038/s41598-019-47176-7, published online 23 July 2019

The access details under the ‘Data Availability’ section of this Article are outdated:

“All behavioural, eye-tracking and neuroimaging data (with personal information de-identified) have been made available on OpenNeuro (https://openneuro.org/datasets/ds001980/versions/1.0.1) and on the PI’s lab website (https://blclab.org/).”

should read:

“All behavioural, eye-tracking and neuroimaging data (with personal information de-identified) have been made available on OpenNeuro (https://openneuro.org/datasets/ds002247/versions/1.0.0) and on the PI’s lab website (https://blclab.org/).”

