# Supplementary material for: Neurocognitive Signatures of Naturalistic Reading of Scientific Texts: A Fixation-Related fMRI Study
Source: Sci Rep. 2019 Jul 23;9:10678. doi: 10.1038/s41598-019-47176-7 (PMC6681173; doi:10.1038/s41598-019-47176-7)
Supplement: Supplementary file 1 — Supplementary Information [file 41598_2019_47176_MOESM1_ESM.docx]

**Neurocognitive Signatures of Naturalistic Reading of Scientific Texts:**

**A Fixation-Related fMRI Study**

Chun-Ting Hsu^1,2^, Roy Clariana^3^, Benjamin Schloss^1^, Ping Li^1*^

^1^Department of Psychology and Center for Brain, Behavior, and Cognition, Pennsylvania State University, University Park, PA 16802, USA

^2^Kokoro Research Center, Kyoto University, 46 Yoshidashimoadachi-cho, Sakyo-ku, Kyoto, 606-8501 Japan

^3^Department of Learning and Performance Systems, Pennsylvania State University, University Park, PA 16802, USA

* Corresponding Authors: [pingpsu@gmail.com](mailto:pul8@psu.edu) (Ping Li)

# **S1: Five Texts Participants Read**

Mars

Could humans live on Mars some day? Scientists ask this question because Earth and Mars are similar. Similar to Earth’s day, Mars’s day is about 24 hours long. Also, both planets are near the Sun in our solar system. Earth is the 3rd planet and Mars the 4th planet from the Sun. Mars also has an axial tilt similar to Earth's axial tilt. An axial tilt gives both planets seasons with temperature changes. Just like Earth, Mars has cold winters and warmer summers. Like Earth, Mars has winds, weather, dust storms, and volcanoes. But in some ways, Earth and Mars are different. Differences include temperature, length of a year, and gravity. The average temperature is -81º F on Mars, but 57º F on Earth. A Martian year is almost twice as long as an Earth year. Earth’s gravity is almost 3 times stronger than Martian gravity. Given the similarities, can humans go to Mars and live there? NASA scientists want to answer this question. NASA oversees U.S. research on space exploration. NASA scientists send devices called spacecraft to explore Mars. The spacecraft carry rovers that can rove or move around. These wheeled rovers can explore characteristics of the planet. They can also show land characteristics and weather on Mars. One of these NASA rovers is named Curiosity. Curiosity found evidence that soil on Mars contains 2% water. NASA has planned a new mission called Mars 2020. This mission will use a new car-sized rover to examine Mars. The new rover will contain additional instruments to study Mars. For example, one instrument will take images beneath Mars’s surface. Another instrument will attempt to make oxygen from carbon dioxide. Mars 2020 will help scientists answer important questions. It will explore whether there has been life on Mars. It will also answer whether humans can live on Mars in the future.

Supertanker

How can engineers help prevent spills of oil from supertankers? Supertankers are huge ships that carry oil over the oceans. A supertanker can contain about a half-million tons of oil. A supertanker is the size of about five football fields. A supertanker’s cargo area could hold the Empire State Building. Most of the world’s oil is transported by these supertankers. Disasters occur when wrecked supertankers spill oil into the ocean. As a result of these oil spills, the environment is damaged. In 1967, a supertanker crashed near the shores of England. This crash resulted in washing ashore 200,000 dead seabirds. In 1989, another supertanker spilled oil into Alaska’s coast. The spilled 11 million gallons of oil caused 1,000 otters to die. Oil spills from supertankers also kill drifting microscopic plants. These plants provide food for sea life, such as whales and shrimp. The plants also produce 70 percent of the world's oxygen supply. Oil spills result partly from limitations in engineering. Supertankers lack double bottom hulls for extra protection. Supertankers lack extra power and steering equipment for safety. Supertankers also have only one boiler to provide the ship power. Supertankers have only one propeller to steer the huge ship. Lack of such backup components causes problems during emergencies. Emergencies for supertankers are ocean storms and coastal reefs. Solutions to problems with supertankers include three tactics. Supertankers must be built with added hulls, boilers, and propellers. These provide extra safety, control, and backup in emergencies. Also, officers need top training to run and maneuver their ships. Supertanker simulators at some facilities provide top training. Finally, ground control stations should be installed near the shore. Ground control stations would act like airplane control towers. They would guide supertankers safely on the oceans along coasts. This ensures safety in shipping lanes and along dangerous coasts.

Math

Two important math concepts are combinations and permutations. Combinations and permutations are similar in several ways. Both refer to sets of objects that you pick and arrange in order. A set could be the numbers 1 through 9, or available pizza toppings. For combinations and permutations, items are selected from a set. For example, we can select 1-3 from the set 1-9 to count and order. However, combinations and permutations are calculated differently. When we compute a combination, the item order does not matter. A combination of 1, 2, 3 is the same as the combination of 3, 2, 1. Another example of a combination is picking pizza toppings. Suppose you pick three toppings: cheese, pepperoni, and sausage. You can also say that you pick sausage, pepperoni, and cheese. Here you have a combination of three things in two different orders. But the pizzas you get are the same because the order doesn’t matter. However, order does matter when we compute a permutation. 1, 2, 3 and 3, 2, 1 are different permutations of these three numbers. In permutations different orders of items have different meanings. Imagine you can pick only one for lunch: pizza, pasta, or rice. You say pizza is your favorite and rice is your least favorite. So your order of preference is: pizza, pasta, and rice. If pizza runs out, the waiter will give you pasta and not rice. But your friend may have a different order of preference. Your friend’s order could be: rice, pasta, and pizza. So two orders of the same items mean something quite different. Permutation allows us to count all the possible orders of items. Given three numbers 1, 2, 3, we can derive 6 permutations. These include 1,2,3 / 1,3,2 / 2,1,3 / 2,3,1 / 3,1,2 / 3,2,1. These sequences in different orders are different permutations.

GPS

Global Positioning System (GPS) is a system that helps us navigate. GPS is a system of 24 or more satellites in space. These satellites orbit at about 12,000 miles above the Earth. Different satellites orbit the Earth in different locations. They circle the Earth along 1 of 6 orbits continuously. They send information to a GPS receiver on Earth. The information is transmitted across space via radio signals. Radio signals travel through space like sound waves through a canyon. Imagine you and your friend are at different sides of a canyon. You shout to your friend and she hears you after a short delay. This delay is the time that sound waves need to reach the other side. Similarly, the satellite in space sends a radio signal at one time. After a delay the radio signal arrives at the GPS receiver on Earth. The receiver records the precise time when the radio signal arrives. It then calculates the difference between these two times. This time difference is the travel time of the radio signal. The GPS uses this travel time to figure out how far the satellite is. It uses the formula “distance = time (T) x rate of transmission (C)”.T is the travel time of the radio signal. C is the speed of light, more than 186,000 miles per second. T x C calculates the distance between the receiver and each satellite. Different satellites have different distances from the receiver. This information helps the receiver determine its location on Earth. The precise location is calculated based on geometry of distances. A GPS device that you carry in your car is a small receiver. Radio signals from the satellites are updated as the device moves. At least 4 satellites are involved to pinpoint the device’s location. GPS devices provide maps and directions that help people travel.

Electric Circuit

Engineers design electrical circuits to power homes and buildings. Electrical circuits are closed paths that electricity flows through. This means that the entire path must be connected like a loop. Imagine a simple circuit made with a battery and a piece of wire. Now connect each end of the wire to different ends of the battery. Your piece of wire and battery now make a circle or circular path. One end of the battery is positive and the other end negative. Electrons will flow across this circuit through the wire. You have just created a simple electrical circuit. Removing the wire from the battery will stop the flow of electrons. Now imagine that you want to connect the battery to a lightbulb. First the wire is cut in the middle and breaks the circuit. Then a lightbulb is inserted to reconnect the circuit. The electrical circuit is complete again and electrons are flowing. Electricity now flows through the lightbulb, lighting it up. If the circuit is now broken, the lightbulb will go out. You can also add a switch in the circuit to break it or connect it. This allows you to turn on or turn off the lightbulb. A simple electrical circuit contains several parts. These include a source, a path, and a resistor. The source provides the energy to an electrical circuit. In our example above, the source is the battery. Batteries can differ in voltage, the electric potential or force. The path is the closed loop with wires that connect to the source. Electrons flow through the path as electric currents. Electric currents increase with increased voltage. The resistor is any device that reduces the electric current. It creates resistance or impedance in the electrical circuit. In our example above, the resistor is the lightbulb. Electric currents decrease with increased resistance.

**S2. Assessment Questions** (Note: correct answer keys in boldface letter)

Mars

1) Compared to Earth's day, Mars' day is:

A. Longer

B. Shorter

**C**. The same

2) Which of the following is true?

**A**. Earth is closer to the Sun

B. Mars is closer to the Sun

C. Mars and Earth are equally distant from the Sun

3) What effect does a planet’s axial tilt have?

A. It allows planets to have water

**B.** It allows planets to have seasons

C. It allows planets to revolve

4) Like Earth, Mars also has:

**A**. Dust storms

B. Waterspouts

C. Rain storms

5) The temperature on Mars is:

A. Warmer than Earth

**B.** Colder than Earth

C. The same as Earth

6) Why is the ability to make oxygen from carbon dioxide important?

A. It may allow us to create water

B. It may allow us to create fuel

**C.** It may allow us to one day live on Mars

7) Compared to Mars, Earth’s gravity is:

**A**. Stronger

B. Weaker

C. The same

8) Why are similarities between Earth and Mars important?

A. They help us learn more about Earth

B. They allow scientists to understand when Mars was formed

**C.** They may make human travel to Mars possible

9) The soil on Mars contains approximately how much water?

A. 1%

**B.** 2%

C. 5%

10) Compared with prior rovers, the rover in the *Mars 2020* mission will:

**A**. Contain additional instruments

B. Be smaller than prior rovers

C. Have the ability to move underground

Supertanker

1) What is the main problem described in the passage?

A. Huge ships crashing into whales

**B.** Supertankers spilling oil into the ocean

C. Airplane control towers are unavailable

2) Approximately how much oil can a supertanker hold?

**A**. A half-million tons

B. Two million tons

C. Three million tons

3) About how large is the average supertanker?

A. The size of a small town

**B.** The size of five football fields

C. The size of the empire state building

4) Why are supertankers important?

A. They travel all over the world

B. They have large cargo areas

**C.** They transport most of the world's oil

5) Why is microscopic plant life important:

**A**. It produces 70% of the world's oxygen

B. It provides 70% of food for people.

C. It helps locate where oil spills occur

6) Which of the following contributes to oil spills by supertankers?

A. Poor communication from ground control stations

B. A lack of officers operating the ships

**C.** Problems with supertankers' engineering

7) What function do double bottom hulls serve for supertankers?

**A**. They provide protection to the supertanker

B. They power the supertanker

C. They help steer the supertanker

8) Why do power and steering problems occur with supertankers?

A. Rain falls heavily during ocean storms

**B.** They have only one boiler and propeller

C. They lack double bottom hulls

9) Which of the following can help to solve the problem of oil spills:

A. Increase the size of the propeller for each supertanker

**B.** Improve training for officers of supertankers

C. Ban travel of supertankers in stormy months

10) What is one benefit of using ground control stations to improve safety?

**A**. They guide supertankers along dangerous coasts

B. They get rid of dangerous coral reefs

C. They provide covered docks for supertankers during storms

Math

1) In what way are combinations and permutations similar?

A. They both deal with order in the same way

**B.** They both refer to sets of objects that are selected

C. They are both calculated in the same way

2) When calculating a combination:

A. Order is important

**B.** Order is not important

C. Order depends on the type of set

3) Which of the following is a combination of 1, 2, 3?

A. 2, 4, 6

B. 4, 5, 6

**C.** 3, 2, 1

4) Choosing pizza toppings is an example of a combination because:

A. The order of toppings changes the pizza

**B.** The order of toppings does not change the pizza

C. The order of toppings depends on the set of toppings

5) Why is order important when calculating a permutation?

A. Different orders have the same meanings

**B.** Different orders have different meanings

C. Different orders make calculation difficult

6) Which calculation allows you to count all possible orders of items?

**A**. Permutation

B. Combination

C. Selection

7) How many permutations can be calculated from the numbers 2, 3, 4?

A. 4

B. 5

**C.** 6

8) Which of the following is a permutation of 3, 4, 5?

A. 5, 5, 3

B. 3, 3, 4

**C.** 4, 3, 5

9) Sequences in different orders form:

A. Different combinations

**B.** Different permutations

C. Different selections

10) Which of the following is true about combination and permutation?

**A**. Combination uses the same items.

B. Permutation uses different items.

C. The same set of items can be either combination or permutation.

GPS

1) How many satellites make up the GPS system?

A. 8 or more

B. 12 or more

**C.** 24 or more

2) Approximately how many miles above Earth do GPS satellites orbit?

A. 8,000 miles

B. 10,000 miles

**C.** 12,000 miles

3) Based on the passage, each satellite:

**A**. Follows 1 orbit

B. Follows 3 orbits

C. Follows 6 orbits

4) Through what type of signal is GPS information sent to GPS receivers?

A. Video signals

**B.** Radio signals

C. Infrared signals

5) Why is the time difference from send to arrival of a signal important?

**A.** It allows a GPS device to calculate the distance of a satellite

B. It allows a GPS device to calculate how fast a satellite is moving

C. It allows a GPS device to update its settings

6) In the formula, *T* x *C*, what does *T* represent?

A. Thrust

**B.** Time

C. Speed

7) The formula *T* x *C* calculates which of the following?

A. Location

B. Speed

**C.** Distance

8) Satellites orbit the Earth along one of how many orbits?

**A.** 6

B. 10

C. 14

9) Signals from how many satellites are needed for a GPS device to work?

A. At least 2

B. At least 3

**C.** At least 4

10) Which of the following information does a GPS device provide?

A. Weather and climate

B. News and reviews

**C.** Maps and directions

Electric Circuit

1) A closed path in a circuit indicates which of the following?

A. The path has a disconnect

**B.** The entire path is connected

C. The entire path is not working

2) Which of the following describes a battery?

A. It has two negative ends

B. It has two positive ends

**C.** It has one positive and one negative end

3) Electrical current refers to which of the following?

**A.** Electrons flowing through a circuit

B. Protons flowing through a circuit

C. Neutrons flowing through a circuit

4) Which of the following is an example of a simple circuit?

A. Two wires connected to the positive end of a battery

B. Two wires connected to the negative end of a battery

**C.** One wire connected to each end of a battery

5) Adding a switch to an electrical circuit allows:

A. The current to change directions

**B.** The current to be turned on and off

C. The current to increase in voltage

6) Which of the following provides energy to an electrical circuit?

**A.** The source

B. The path

C. The resistor

7) Electrical current decreases as:

**A.** Voltage decreases

B. Voltage increases

C. Voltage stays the same

8) Changes in the amount of voltage are based on:

A. Changes in a circuit’s source

B. Changes in the path of a circuit

**C.** Changes in electrical potential

9) A battery in a simple electrical circuit serves as:

**A.** The source

B. The path

C. The resistor

10) The amount of electric current increases as:

A. Resistance increases

**B.** Resistance decreases

C. Resistance stays the same
